# Supplementary material for: Chaos in synthetic microbial communities
Source: PLoS Comput Biol. 2022 Oct 10;18(10):e1010548. doi: 10.1371/journal.pcbi.1010548 (PMC9584520; doi:10.1371/journal.pcbi.1010548)
Supplement: Attachment — Submitted filename: Response-to-Reviewers.docx [file pcbi.1010548.s002.docx]

**Reviewer #1:**

Here Karkaria and colleagues present a new method for exploring chaos within a in silico microbial communities. They apply this method to one particular family of in silico models (composed of 3 species, 2 QS systems, 3 bacteriocins) to identify a small subset of models in which chaos is most likely. They then explore in more depth how chaos depends upon parameter sets for one specific model, m850, identified by their initial screening process.

Overall, this paper reads as a thorough explanation of chaos in one particular in silico model. It’s well written, the figures are helpful, and the models were largely well explained - however, my impression is that the focus seems far too narrow to be of interest to the broad plos comp bio readership.

We thank the reviewer for the positive comments on the manuscript. We accept the criticism that the way we have presented our new methodology, it is unclear what the goals of the paper are.

Whether a paper is of sufficient interest for the journal is up to the editor. However, I will try and outline the sort of thing I would expect to see to make this paper more broadly applicable, depending on what exactly the authors were aiming to achieve. For example,

a. Was the goal to present a new method for identifying the likelihood of chaos within microbial communities, ABC SMC? If so, I’d have expected to see some proof that it works – ie are there any empirical data out there the model can be compared against? Or, at the very least, I would expect to see the method applied to a far broader set of models (more species etc) to explore how scalable it is.

b. Alternatively, was the goal to build a general conceptual understanding of what shapes chaos within microbial communities? If so, again I’d expect to see a far broader exploration of different microbial systems – ie different numbers of species, different interaction types etc etc. Even just within the 3 species, 2QS model presented, I’d want to know whether the the parameter relationships seen in m850 hold for other model formulations. I would also want more of a discussion of the biological relevance, ie how do the patterns observed compare to what we see in nature, or in existing (in vitro) synthetic communities?

c. Or, was the goal to really dig into the behavior of m850? If so, here I’d definitely expect to see some comparison against empirical data – for example experiments altering dilution rates and showing transitions to chaos.

The goal of the paper is to present a novel methodology for exploring chaotic behaviour (option a above) and use this new methodology to explore the presence of chaos in small, engineered consortia, which has not been explored previously. We chose a recently developed suite of synthetic microbial communities for which we have mechanistic and parametric understanding.

The results are critically important for the fields of synthetic biology and metabolic engineering where a major focus of effort is on constructing small microbial communities for bioprocesses. By showing that chaotic behaviour arises in systems that could easily be built today, we have demonstrated that chaotic behaviour needs to be accounted for when designing communities. i.e. that it is a real, rather than a theoretical, concern. This is also why the paper is of interest to a wide range of interdisciplinary researchers and, we believe, entirely suitable for publication in PLoS Computational Biology. We have amended the title of the manuscript to reflect and emphasis this focus.

In order to make this clearer to the reader, we have added in a new section and Figure 1 where we explain the novel methodology in more detail and prove its applicability in a four-species generalized Lotka-Volterra model previously explored. This not only shows that the algorithm agrees with previous results obtained through brute-force sampling but also shows how we can quantify the parameter regions that give rise to chaotic behaviour, identifying parameter combinations that are sensitive. Since this is done in multidimensional space it complements standard continuation methods and bifurcation analyses.

Smaller comments

Pg 2 “While these abstractions are suitable in some circumstances, using them to inform gene regulation networks and community design can be difficult” – why?

We agree that this was a confusing statement. We have modified the text as follows:

“While these abstractions are suitable in some circumstances, by modelling the intermediates involved in competitive interactions we can include experimentally measurable mechanisms and parameters.”

2.2 “Here we see some evidence for a peak in…” this seems like an interesting result relative to past work, picking up the theme of how generalizable are your results – would have been nice to explore further.

Para 1 page 7, this section wasn’t super clear, perhaps needs stepping through a bit how these results compare to previous literature.

Both these points are addressed in more detail later in this section. We have added some text to put it into context with our previous work.

“Cooperative interactions were previously found to give rise to unstable systems, whereas competition was more indicative of stability [26]. The same effect might occur here in systems with one QS, rather than two, as the system would be expected to have increased correlation. While chaotic behaviour appears to be very different from linear stability, both behaviours share the necessity for coexistence. Our previous work showed that SL interactions were important for producing linear stability [17]. This may explain why we see tendencies for topologies to share a mixture of stability associated SL interactions, and instability associated OL interactions.”

Discussion “We show that we can expect to find chaotic states in relatively small synthetic microbial systems, which has important ramifications for the field” Coming back to my concerns about novelty, we already know you can find chaos in 3 species systems, I’m not sure the distinction between natural vs synthetic is super meaningful, so where really is the novelty?

As stated above, I think this result is critically important for the fields of synthetic biology and metabolic engineering. We have demonstrated that chaotic behaviour needs to be accounted for when designing novel communities.

“Although chaotic attractors are generally thought to be sparse in low dimensional systems, we have shown their existence in realistic synthetic microbial systems.” Are they not also sparse in your system 25/4000 something? Or are they far more sparse in your system than others?

Actually, when considering the space of oscillators, the results are not sparse; roughly ¼ of the oscillating systems demonstrated chaos. However, the point is that chaos can arise in systems that people are constructing in the context of metabolic engineering. This moves the issue from mathematical possibility into actual design considerations.

**Reviewer #2:**

The authors develop and apply methods for for identifying chaotic parameter regimes using ABC SMC, and thereby identifying models/model topologies that permit chaotic behaviour. This is applied in the context of models of small microbial communities governed by resource competition, intercellular communication and competitive bacteriocin interactions. The inference and "search for desired behaviour" aspects seem to build upon the corresponding author's 2011 PNAS paper (an observation that is not intended as a criticism, and I would stress that the present manuscript is certainly not lacking in novelty). I found the manuscript to be interesting and generally clearly written (and the Zenodo repo is also appreciated), and have included a few points and suggestions below that I hope the authors may find useful.


Minor points:
==========

0. There is an additional key paper that I think the authors should cite, which I suspect has been inadvertently omitted (unless I missed it): Barnes, C. P., Silk, D., Sheng, X., & Stumpf, M. P. (2011). Bayesian design of synthetic biological systems. Proceedings of the National Academy of Sciences, 108(37), 15190-15195.

Thank you for the suggestion, we have now added a reference to this paper in the results section where we first introduce the use of ABC SMC.

1. The abstract could be more specific, which may help to encourage a larger readership for the manuscript. In particular, a brief description of the "methodology to explore the potential for chaotic dynamics" would be appreciated, mentioning ABC and the "filtering" approach of looking for oscillations and then chaos.

This is a great point, we have adjusted the abstract accordingly by adding the following statement:

“Our model selection pipeline uses Approximate Bayesian Computation to first identify oscillatory behaviours as a route to prioritising our ability to find chaotic behaviour”

2. General comment: when talking about "posterior probabilities", I think it needs to be made clear that these are posterior probabilities *given* the desired/target qualitative behaviour (to distinguish from the more usual situation where they would be conditioned on an observed dataset). Perhaps this need only be done once, the first time the expression is used, to make clear what is meant.

We have added some statements to be more explicit about what the posterior distributions mean, and the connection between the oscillatory objective posterior and chaotic objective prior

“Models identified as capable of producing oscillations become the prior model distribution for the next stage”

“The posterior probabilities of the models for the chaotic objective and the prior distributions used are shown in Figure 2b”

3. Caption for Fig 1: Spell out what A_i, B_j and N_k denote in panel (a). In the second and third boxes of panel (a), could the criteria for oscillation and chaos be briefly summarised, e.g. for oscillations, even just listing the three d_{oi} and then referring forward in the caption to the Methods, would help to guide the reader. For (c), I'm not sure the y-axis "OD 600" is clearly explained, and I was unclear what was meant in the caption by "An example time series representative of the dataset" (which dataset?). Finally, I don't think the figure at the bottom panel c is explicitly described in the caption or text?

Thank you for the suggestions, we have added the following statements for clarity on this Figure:

Added to section 2.2:

“Three strains, N1,N2,N3, optionally express bacteriocins ,B1,B2, under the control of optionally expressed QS molecules A1,A2. The QS molecules regulate expression of bacteriocins positively or negatively. Each strain can be optionally sensitive to a bacteriocin. The initial model space describes an enumeration of possible combinations of bacteriocin and QS systems, and forms the first uniform prior model space “

Figure 2 Caption changes:

“We then perform ABC SMC for an oscillatory objective, where simulations produce λ1 > 0.003, which yielded 117 models that were capable of producing oscillations.”

“Population densities shown as optical density (OD) show sustained, nonrepetitive oscillatory behaviour for the three species community.”

“An example time series representative of the chaos objective posterior distribution. Population densities shown as optical density (OD) show sustained, nonrepetitive oscillatory behaviour for the three species community.”

4. Is Algorithm 2 (ABC SMC) referenced in the text? Apologies if I missed it. In this algorithm there is a single distance, d (line 15 of algorithm), whereas for the "Oscillatory population dynamic objective" there are three distances. How does d relate to these three distances?

Thanks for noticing this. We have added a reference to the algorithm at the start of Section 2.1 (now Algorithm 1) We also added a reference to Algorithm 2 in this section, that describes how we calculate the maximal Lyapunov exponent.

For each objective we have now defined distances as a set so that it relates back to the ABC SMC algorithm correctly. Some text in the methods section has been added to explicitly note how multiple distances are handled in accepting and rejecting particles.

“Particles are first sampled from the prior distribution and simulated. A set of distances, d, are calculated from the simulation. If all distances are less than the intermediate threshold, εt, the particle is accepted (d < εt ).”

5. Caption for Fig 2: "The bar chart shows the mean model posterior probability across three experiments, represented by the scatter points..." Apologies, I may be missing something here: to what does "the scatter points" refer?

Thank you for pointing this out. This statement has been removed and the figure caption amended

6. Near start of Section 2.2, stating that m_k denotes the k-th considered model would aid the reader.

We agree this would help readability and have added the reviewer suggestion.

7. Prior model probabilities: I'm not sure if these were stated anywhere (apologies if I missed it). Presumably uniform over all considered models?

That is correct, we use a uniform prior at each stage of the pipeline. We have added some statements for clarity in section 2.2

“The initial model space describes an enumeration of possible combinations of bacteriocin and QS systems, and forms the first uniform prior model space”

“We identified 117 models capable of producing oscillations. These models become the new uniform prior model distribution for the next stage, …”

8. "The classic debate on the complexity-stability relationship in theoretical ecology is likely highly dependent on the nature of the biological interactions involved". This is an interesting point, which several authors have considered, e.g. (i) Allesina, S., & Tang, S. (2012). Stability criteria for complex ecosystems. Nature, 483(7388), 205-208; and (ii) Kirk, P., Rolando, D. M., MacLean, A. L., & Stumpf, M. P. (2015). Conditional random matrix ensembles and the stability of dynamical systems. New Journal of Physics, 17(8), 083025.
Do the authors believe that some of the "design principles" for stability (e.g. the stability criteria described by Allesina and Tang) could provide helpful prior information, e.g. to help restrict the set of models that is considered?

It could be something we consider in the future. We felt the initial oscillatory objective ABC SMC would go some way to reducing the model space in search of chaos. I can see where we could add an additional step before the oscillatory objective to improve the efficiency of the pipeline

We have added this section to the discussion, together with references to Allesina & Tang and Kirk et. al:

“By first running ABC SMC for the oscillatory objective we are able to drastically reduce the model space we are searching for chaos. However, we the sparsity of chaos makes this pipeline computationally costly and reduces the resolution of the posteriors we can achieve in a given time frame. For the future we will consider using eigenvalue stability methods to reject particles without simulation , improving the efficiency of our pipeline and therefore improving the posterior resolution [31,32]”

9. In Section 2.3, when the random forest classifier is introduced, it took me a little while to understand what was being done. I appreciate that the details are provided in the Methods, but I felt that perhaps the definition of the training and test sets needed to be briefly provided in Section 2.3, to aid comprehension. It would moreover seem appropriate to cite Breiman. Perhaps also some of the ABC-RF work (e.g. Pudlo, P., Marin, J. M., Estoup, A., Cornuet, J. M., Gautier, M., & Robert, C. P., 2016), if the authors feel it would be appropriate.

Here we’re not using random forest as part of model selection or parameter inference process, we’re using it as a tool to assess the importance of parameters that can distinguish between an oscillatory objective posterior and a chaotic objective posterior in model 850. The train test split was done to ensure the model is learning some generalisable rules as opposed to learning the dataset.

We added some text to be more explicit in the results section about how this random forest train/test is setup:

“We curated an label-balanced dataset that consisting of input vectors that produced either oscillations, or chaos}. Using a train test ratio of 0.5, the trained classifier model was able to classify the test set with a 90\% accuracy (Methods, Figure 6).”

10. Given the definition of chaos, it might be worth briefly commenting on the limitations of fixed precision ODE solvers, such as the one used here. This is not an area about which I know a great deal, but I would direct the authors toward, for example: Hu, T., & Liao, S. (2020). On the risks of using double precision in numerical simulations of spatio-temporal chaos. Journal of Computational Physics, 418. Exploring this in detail would be beyond the scope of the current manuscript, but perhaps a comment to say that this could be worthy of further investigation (if the authors agree that it might be?).

Anecdotally, we don’t appear to have issues with reproducing chaotic trajectories when using the same parameters, suggesting the fixed precision does not have a qualitative effect for the amount of time we are simulating for. Furthermore, for the purpose of this paper we are only interested in whether a trajectory behaves chaotically or not, rather than reproducing the trajectory. I think the paper you refer to is interesting and would certainly have implications if we were looking to reproduce a specific chaotic trajectory.

11. Figure 3d -- the green dots are a little difficult to see. Could a different marker shape be used? Figure 3c -- include units (bits) on the y-axis.

Thank you for the suggestions. In Figure 3d we changed green dots to black stars to make them more distinctive and Figure 3c has been amended to include the units

12. I was unable to run the Matlab p files provided in the repo, receiving an error: "Invalid pfile. The file header is corrupt." This may just be my system (I did not have chance to look into the error in any depth), but I would be very grateful if the authors could look into this (even if the conclusion of their investigation is that this is a problem that is specific to this reviewer, in which case they can just throw this back to me to investigate).

The scripts from the repo should be run with python and can be used to produce the figure assets from the raw data provided in the repo. If you have any other problems getting this working, please let us know.

Typographical and grammatical points:

Thank you for taking the time to spot and compile these. We have fixed these errors.
