# Supplementary material for: Chaos in synthetic microbial communities
Source: PLoS Comput Biol. 2022 Oct 10;18(10):e1010548. doi: 10.1371/journal.pcbi.1010548 (PMC9584520; doi:10.1371/journal.pcbi.1010548)
Supplement: Attachment — Submitted filename: Response-to-reviewers.docx [file pcbi.1010548.s003.docx]

Reviewer #1: The authors have adequately addressed all of my previous comments. I particularly appreciate the inclusion of the 4 species gLV model which helps with both the generality and the understanding of their method.

Thank you for your suggestions in the review process. We think these have been really useful in improving the impact of our manuscript.

Small comments:

I am still curious about the peak in probability of chaos with increasing number of parts (line 127) - is there any way of determining whether this is real and not an erroneous prediction of your model (eg unbiasedly sampling from models with varying number of parts and comparing their stability within simulations?)

Each model is treated independently. In Figure 3 we are showing the best performing model when subsetting for the number of parts. That is not to say that all models with 4 parts perform better than all models with 5 parts. All models have the same prior probability, meaning they have an equal probability of being sampled, simulated and tested. Additionally, the error bars for the model posterior probability on Figure 3a suggest we have taken a sufficient number of samples to draw conclusions from this plot.

However, the conclusions we are drawing here are limited to the prior that we use. We have added this section of text to draw attention to this.

“We highlight that these observations are limited to the small communities defined in our prior. These properties may not be reflective of larger communities, however, we hypothesise that a trade-off between stabilizing interactions that enable co-existence, and destabilising interactions to prevent linear stability, will remain important for producing chaotic population dynamics.”

More importantly I do not think the comparison to the complexity-stability debate is entirely appropriate here, given this typically refers to complexity in terms of number of species or number of interactions, not complexity of the interactions themselves. I would rephrase this section to make this distinction much clearer.

We think this is a fair comment. We have rephrased this section to focus more on the contribution negative interactions make to producing linear stability.

“Our previous work demonstrated that system stability increased with the number of parts. The peak in the posterior probability for chaos at four parts reflects a balance that includes enough mechanisms to enable co-existence, without the tighter network of negative interactions that are associated with stable steady state.”

Reviewer #2: Thanks to the authors for addressing my points. I have only very minor comments remaining:
1. I still think it would be appropriate to cite Breiman re: random forests; namely, Breiman, L., 2001. Random forests. Machine learning, 45(1), pp.5-32.

Thank you for the suggestion, we agree this is appropriate and have added the citation.

2. I think the figure numbers in the README of the Zenodo repo may now be out of sync with the manuscript (but I can understand the authors not wishing to update these until the end of the review process, to avoid having to repeatedly update the repo).

Yes, we will update the Zenodo once revisions are completed
